# Animal Models for Mechanical Circulatory Support: A Research Review

**DOI:** 10.31083/j.rcm2510351

**Published:** 2024-09-29

**Authors:** Buyan-Ochir Orgil, Michelle Chintanaphol, Neely R. Alberson, Lea Letourneau, Hugo R. Martinez, Jeffrey A. Towbin, Enkhsaikhan Purevjav

**Affiliations:** ^1^The Heart Institute, Department of Pediatrics, University of Tennessee Health Science Center, Memphis, TN 38103, USA; ^2^Children’s Foundation Research Institute, Le Bonheur Children’s Hospital, Memphis, TN 38103, USA; ^3^College of Medicine, University of Tennessee Health Science Center, Memphis, TN 38103, USA; ^4^Rhodes College, Memphis, TN 38112, USA; ^5^Pediatric Cardiology, St. Jude Children's Research Hospital, Memphis, TN 38105, USA

**Keywords:** animal model, mechanical circulatory support, left ventricular assist device, heart failure, mechanical unloading

## Abstract

Heart failure is a clinical syndrome that has become a leading public health problem worldwide. Globally, nearly 64 million individuals are currently affected by heart failure, causing considerable medical, financial, and social challenges. One therapeutic option for patients with advanced heart failure is mechanical circulatory support (MCS) which is widely used for short-term or long-term management. MCS with various ventricular assist devices (VADs) has gained traction in end-stage heart failure treatment as a bridge-to-recovery, -decision, -transplant or -destination therapy. Due to limitations in studying VADs in humans, animal studies have substantially contributed to the development and advancement of MCS devices. Large animals have provided an avenue for developing and testing new VADs and improving surgical strategies for VAD implantation and for evaluating the effects and complications of MCS on hemodynamics and organ function. VAD modeling by utilizing rodents and small animals has been successfully implemented for investigating molecular mechanisms of cardiac unloading after the implantation of MCS. This review will cover the animal research that has resulted in significant advances in the development of MCS devices and the therapeutic care of advanced heart failure.

## 1. Introduction

Heart failure is the clinical syndrome and final pathway from a myriad of cardiovascular diseases that result in inadequate heart muscle pumping of blood to meet the body’s needs [1, 2]. Nearly 64 million individuals are currently affected by heart failure worldwide, presenting a significant public health problem in both developed and developing countries [3, 4, 5]. The 5-year heart failure mortality rate is estimated as 50%, which increases to nearly 90% for the 10-year risk prediction [6]. Left ventricular assist devices (LVADs) are designed to mechanically support failing circulation in patients with acquired or congenital end-stage heart failure as bridge-to-transplant therapy (BTT), bridge-to-recovery, bridge-to-decision, or destination therapy [7, 8]. The short-term survival rates for adult patients undergoing LVAD implantations are as high as 90% at 6 months and 79% at 24 months [9], while newer models of LVADs with post-implant survival as 51% at 7 years aim for long-term support [10, 11]. Patient selection and implantation timing can be simplified by incorporating the Interagency Registry for Mechanically Assisted Circulatory Support (INTERMACS) profile as a reference to categorize patients who are at a higher risk for morbidity and mortality with lower INTERMACS profiles [8]. In this classification, patients who are inotrope-dependent (INTERMACS profiles 2 and 3) are suitable candidates for chronic LVAD support [12].

Research using animal models is unquestionably important for developing medical devices that are intended to be used in humans [13, 14]. This review focuses on animal models for preclinical mechanic circulatory support (MCS) studies that have been pursued in many laboratories (Table 1, Ref. [15, 16, 17, 18, 19, 20, 21, 22, 23, 24, 25, 26, 27, 28, 29, 30, 31, 32, 33, 34, 35, 36, 37, 38, 39, 40, 41, 42, 43, 44, 45, 46, 47, 48, 49, 50, 51, 52, 53, 54, 55, 56, 57, 58, 59, 60, 61, 62, 63, 64, 65, 66, 67, 68, 69, 70, 71, 72, 73, 74, 75, 76, 77, 78, 79, 80, 81, 82]). The summarized animal studies include research on the development and improvement of new MCS devices, their surgical methods and training, investigating the physiology of the left ventricular (LV) and right ventricle (RV) unloading, underlying mechanisms of cardiac remodeling during MCS support, LVAD complications, and regerative therapies with the focus on clinical implications such as therapies of heart failure and myocardial ischemia (MI), major causes of morbidity and mortality worldwide [83]. In addition, due to present challenges in pediatric MCS and post-LVAD management, we included a review of animal models utilized for the development and improvement of LVADs intended for use in pediatric patients with advanced heart failure.

**Table 1.** **Animal modeling in ventricular assist device development and reseacrh**.

Research aims	Animal models [references]
Device development, performance, durability and bicompatibility	Bovine [15, 16, 17, 19, 64, 65]; Ovine [24, 25, 77, 79]; Porcine [72, 82]
Surgery techniques and training	Bovine [18, 65]; Porcine [41, 49, 66, 67]; Goat [69]
Device comparison	Bovine [21, 22]; Ovine [30, 42, 43, 47, 78, 82]; Porcine [72, 82] Canine [52, 53, 54, 55, 56]; Goat [69]
Miocardial ischemia modeling	Ovine [26, 28]; Porcine [33, 34, 35, 36, 37, 38, 39, 40, 43, 44, 45, 46, 47, 48]; Canine [50, 51]
Physiological responces to LVAD	Ovine [29]; Porcine [32, 42, 43, 47, 49]; Canine [51, 52, 56]; Goat [69]; Rat [70]; Ovine [76]
Cardiac remodeling to LVAD	Ovine [27, 28, 29]; Porcine [33, 36, 37, 38, 39, 40, 47, 48]; Canine [51]; Rat [57, 58, 59, 60, 61, 62]
LVAD complications	Bovine [20, 64]; Multiple species [68]; Goat [69]; Rat [70]; Porcine [71, 72]; Ovine [73, 74, 75, 76, 77, 78]
LVAD with vasoactive and regenerative therapy	Bovine [22, 23]; Porcine [43, 44, 45, 46]; Rat [62]
Pediatric LVAD development and use	Porcine [71, 72]; Ovine [73, 74, 75, 76, 77, 78, 79, 80]; Porcine [81, 82]
RVAD and RV failure	Ovive [31]; Goat [69]; Rat [70]; Ovine [80]
Reverse remodeling to unloading using hHT_x_ and engineered heart tissues	Rat [57, 58, 59, 60, 61, 62, 70]; Mouse [63]

LV, left ventricular; RV, right ventricular; AD, assist device; hHT_x_, heterotrophic heart transplant; LVAD, left ventricular assist device; RVAD, right ventricular assist device.

## 2. Large Animal Modeling in Preclinical LVAD Research

The applicability of animal models in LVAD development to clinical practice is limited by species-specific anatomical differences and the species-dependent differences in cardiac remodeling [15]. A review by Monreal *et al*. [84] delineated these differences and summarized the various heart failure-induced animal models used in preclinical LVAD research. Nevertheless, large animals are instrumental in preclinical device research and development and several species of domestic animals have been used for the evaluation of LVAD safety and feasibility. Additionally, large animals are important preclinical studies of MI, mechanical unloading and complications associated with LVAD implantation such as hemorrhagic and thromboembolic events, pulmonary hypertension, arrhythmias, and organ failure [16].

### 2.1 Bovine (Calves) Studies

Calves have similar weight to the human body and large thoracic cavity, allowing for effective cannulation and vascular access. Although calves have a single brachiocephalic trunk arising from the aortic arch, cannulation remains similar in clinical relevance. Therefore, the inflow tract in the bovine model is inserted into the apex of the heart to reach the LV, and the outflow graft anastomosed to the descending aorta [17, 18, 84]. Bovine studies have also offered valuable insights into biocompatibility of LVADs, hemodynamic responses, and potential strategies for optimizing and advancing LVAD performance in patients. Preclinical testing of the HeartMate III, a third-generation compact and centrifugal LVAD, analyzed pump performance, hemodynamic flow, hemolysis, and shear stress exposure in calves [17, 19]. A study by Schenk *et al*. [20] evaluated the use of MagScrew VADs with clot-inhibiting surface in three calves monitored for a time course between 10 and 90 days. Despite a complication of endocarditis of the prosthetic valve and infectious embolization in calves, the device demonstrated feasibility and potentials for long-term functionality [20].

Multiple types of LVADs have been developed based on flow type, pulsatile or continuous. Pulsatile flow VADs (PFVAD) have been associated with an increased risk of thromboembolism and have thus led to the development of continuous flow VADs (CFVAD) [21]. While the latter is associated with a decreased risk of thromboembolism, the mechanical composition of these LVADs still poses risks of device failure and clot formation. One group of researchers developed an LVAD with an axial (AX) flow pump with a hydrodynamic bearings levitation system to address these risks [21]. They implanted the pumps into four calves for up to 90 days and found the device to be biocompatible with a bovine model. Another bovine model evaluated differences between AX and magnetically or hydrodynamically levitated centrifugal LVADs. Eight AX and 11 centrifugal LVADs with different flow rates and pump speeds were placed *via* left thoracotomy in Jersey calves with MI heart failure induced by injecting microspheres into the left anterior descending (LAD) and circumflex coronary arteries. The calves received up to 60 days of LVAD support and researchers performed hemodynamic, electrocardiographic (EKG), and echocardiogram monitoring [22]. Results showed that centrifugal LVADs required less power, demonstrated superior flow estimation, and exhibited steeper curves in hydrodynamic performance, indicating increased sensitivity to preload and afterload changes compared to AX LVADs. However, no significant differences in LVAD flow, end-systolic volumes, end-diastolic volumes, ejection fractions, regional blood flow, or EKG parameters were identified between devices.

Researchers have also tested different parameters in the pump speed of compact turbo rotary blood pump which uses an AX flow pump. Typically, these devices pump blood at a constant speed defined by revolutions per minute (rpm) and lessen vascular pulsatility and variation in ventricular end-systolic and end-diastolic volumes, which may provoke aortic insufficiency and gastrointestinal bleeding. The bovine model of MI heart failure was implanted with a centrifugal-flow LVAD, and different speed modulation profiles were tested and parameters related to heart function, blood flow, and device performance were measured [85]. The study demonstrated that modulation of pump speed augmented aortic pulse pressure and improved cardiac function and end-organ perfusion compared with the constant speed module. The asynchronous mode provided the technological advantage of sensorless control and suggested that speed modulation of MCS may reduce adverse events in patients with LVAD support [85].

To study different hemodynamic responses in CFVAD or PFVAD, Bartoli *et al*. [23] induced either chronic or acute heart failure in 14 male mixed-breed calves and implanted either a CFVAD (HeartMate II) or PFVAD (Thoratec PVAD) in these calves. Results of monitoring blood pressure and recorded flow waveforms demonstrated altered energy utilization profiles in myocardial and vascular hemodynamics in CFVAD treated calves. For example, continuous unloading significantly altered LV peak systolic pressure and aortic systolic and diastolic pressure [23]. In contrast, preserved normal physiological values were demonstrated with the use of a PFVAD.

Lastly, bovine models have been used to study combined LVAD and regenerative therapies to improve restoration of myocardial function. Regenerative therapies include cell or gene therapy, as well as the use of growth factors, exosomes, and biomaterials. These strategies summarized by Tseng *et al*. [86] can potentially be synergistically integrated with LVADs to stimulate greater myocardial recovery than that achievable with either therapy alone. The important effects of MI are infarct size and progressive cardiac fibrosis with imbalanced growth and deposition of extracellular matrix (ECM). A meta-analysis of 26 animal studies involving 488 animals found that infarct size was reduced in LVAD-supported animals compared to non-LVAD controls [24]. Findings also showed that earlier LVAD implantation following acute MI reduced infarct size.

In another study investigated the combination of LVAD and intra-myocardial injections of ECM particulates (P-ECM) in bovine models of chronic MI heart failure [87] and found integration of P-ECM sheets into surrounding tissues that provided a favorable scaffold for myocyte and fibroblast differentiation and proliferation. Results demonstrated that P-ECM injections combined with LVAD support increased ejection fraction, cell proliferation, and end-organ regional blood flow [87].

### 2.2 Ovine (Sheep) Studies

Sheep and humans are alike in body size, cardiac output, valvular anatomy, and the absence of coronary collateral supply [25]. Despite differences in heart positioning in ovine models being quadrupedal with left-dominant coronary supply in contrats to humans are having right-dominant coronary circulation, sheep with many anatomic similarities are suitable and advantageous for preclinical LVAD studies [25, 84]. An ovine model was successfully used for *in vivo* testing of a third-generation centrifugal magnetic suspension LVAD. This device was implanted in six healthy adult sheep for thirty days to monitor hemocompatibility with no evidence of hemorrhagic or thromboembolic complications [88].

Percutaneous MCS (pMCS) devices are small balloon pumps that can be placed in the LV across the aortic valve (intraventricular) or in the descending thoracic aorta (intra-aortic) using catheters through the femoral artery [89]. Given considerable attentions for an aging population and improved heart failure treatments, the use of pMCS devises such as intra-aortic balloon pump (IABP), Impella and extracorporeal membrane oxygenation (ECMO) has expanded as alternative management to cardiac surgery in patients with high surgical risks [26, 27]. One study evaluated the use of pMCS in the descending thoracic aorta in adult Swiss White Alpine sheep that have comparable inner-aortic diameters, heart rates, and cardiac outputs to those in humans. While the device failure was complicated in first four sheep by due to changes in aortic diameter, this problem was resolved in subsequent pMCS device placement with an aortic stent and device function was successfully monitored for 30 hours. The study highlighted that intravascular stents may be needed to replicate the arterial wall stiffness of more elderly patients and demonstrated the safety and biocompatibility of pMCS devices [89].

Ovine models have also been used to investigate effects of mechanical unloading in the setting of acute MI and MI-induced heart failure. A transvalvular microaxial Impella blood pump placed through the aortic valve is a miniaturized rotary blood pump which aspirates blood from the LV and releases it into the ascending aorta [27, 28]. Meyns *et al*. [90] aimed to investigate the effect of a catheter-mounted blood pump on MI size in a sheep model of ischemia-reperfusion injury (IRI). The Impella inserted into 26 Dorset sheep *via* the carotid artery significantly (*p* = 0.00001) reduced MI size in animals tested and diminished myocardial oxygen consumption, which was strongly correlated with the reduction of infarct size (r = 0.9). The reduction in infact size was also associated with the degree of mechanical unloading during IRI [90].

Cardiac apoptosis is a common adverse remodeling that occurs in patients with severe and end-stage heart failure [91]. Ovine studies have yielded important information on cytokines, biomarkers, and gene expression during mechanical unloading after LVAD implantation. The FW-II type AX flow LVAD was implanted in sheep during the early stage of MI [29] and histopathology of the heart following three-day LVAD support demonstrated reduction in number of apoptotic terminal deoxynucleotidyl transferase dUTP nick end labeling (TUNEL)-positive cardiomyocytes, decrease in inflammatory cell infiltration and myocardial fibrosis [29]. Two differentially expressed microRNAs (miR), oar-miR-19b and oar-miR-26a, were identified as being closely associated to reduction of apoptosis, suggesting effects of miRNAs and proinflammatory signaling in heart failure following LVAD implantation [29]. Supporting this data, a high expression of cytokines such as tumor necrosis factor-alpha (TNF-α), caspase-9, interleukins 1 beta (IL-1β), and IL-6 in the heart and serum of LVAD candidates are associated with the severity of heart failure [30, 31]. Wei *et al*. [92] further demonstrated beneficial molecular remodeling in MI model of Dorsett hybrid sheep in response to short-term LVAD unloading. Tightly regulated cardiomyocyte calcium transients play a vital role in cardiac contractility and changes in calcium cycling may lead to cardiac dysfunction in the failing heart [30]. In this study, sheep with sustained MI were mechanically unloaded with the Impella 5.0 (AX-flow LVAD) for 2 weeks and were observed for 12 weeks post-MI [92]. The study demonstrated improved heart function and normalized calcium-handling proteins (CHPs) and Ca^2+^-ATPase (Adenosine TriPhosphatase) activity in the tissue surrounding the MI in sheep undergoing MCS compared to sheep that did not receive LVAD support [29].

Common LVADs operating modes have fixed rate, synchronized to the heart rate, or automatic ejection rate triggered when the pump chamber becomes full [29]. Amacher *et al*. [93] investigated the use of a PFVAD synchronized with the R-wave across ten different phase shifts/ejection delays in an ovine model. These phase shifts were shown to impact various hemodynamic parameters, including an ejection delay of 10% of the cardiac period, resulting in 43% stroke volume (SV) reduction compared with baseline, and a delay of 70% maximum stroke work resulting in an 11% SV increase [93]. Researchers suggested that gradual alterations in hemodynamic parameters may be useful for reloading therapy when patients are tapered off LVAD support.

An ovine model has also been used to investigate benefits of LV unloading for hearts with right ventricle (RV) pressure overload that occurs in several types of congenital and acquired heart diseases [94]. Leeuwenburgh *et al*. [94] performed pulmonary artery (PA) banding in sheep to create RV pressure overload, and LV unloading was done by a total left heart bypass using a centrifugal pump. Although LV unloading decreased RV contractility in both hearts, with or without RV pressure overload, cardiac output was increased in the PA banding group, suggesting that cardiac output was improved likely due to improved RV diastolic function [94].

### 2.3 Porcine (Pig) Studies

Extensive LVAD research has been conducted using porcine models, as the porcine aortic valve, coronary artery distribution and blood supply to the body and organs are similar to humans [32, 33, 34]. Delmas *et al*. [35] evaluated the efficacy of an MCS device, IVAC2L PulseCath, in 6 to 8-month-old male pigs with induced cardiogenic shock (CS). The IVAC2L system was implanted through the aortic valve in the LV and the pigs received device support for a median time of 34 hours. The IVAC2L support increased blood pressure and decreased pulmonary capillary wedge pressure (PCWP) in both healthy pigs and pigs with CS. Cardiac output was increased in pigs with CS, demonstrating the potential feasibility of the device for long-term use [35].

Porcine acute MI models have been used to investigate effects of MCS devises. The pMCS IABP devise was used in Yorkshire pigs subjected to IRI [36] and the study demonstrated that partial LV unloading with an IABP before reperfusion decreases myocardial necrosis compared with either reperfusion alone or LV unloading after reperfusion. The study also demonstrated a correlation between an inhibition of myocardial endothelin-1 (ET-1) release and myocardial protection [36]. In another study examining LV unloading before reperfusion, Esposito *et al*. [37] used a transvalvular AX pump (TV-pump) in adult Yorkshire pigs after inducing acute MI. Results showed that thirty minutes of LV unloading, compared to primary reperfusion, decreased infarct size and LV fibrosis and increased levels of cardioprotective stromal cell-derived factor-1 alpha (SDF-1α) cytokines [37]. A study by Watanabe *et al*. [38] compared LV unloading *via* the Impella LVAD and nitroprusside 2 weeks after induced MI in Yorkshire swine. The researchers found reduced LV end-diastolic wall stress in both the Impella and nitroprusside groups; however, the latter was associated with pharmacological systemic hypotension in 2 of the 4 pigs. End-diastolic wall stress was associated with impaired microvascular perfusion, suggesting that LV unloading with an Impella may also increase microvascular perfusion of the infarcted myocardium [38].

Acute MI has been shown to cause to not only LV function impairment but also hemodynamic changes of pressure-volume overload in the left atrium (LA), resulting in myocardial stretch of both chambers [39, 40]. A study by Ishikawa *et al*. [39] aimed to define the effect of acute changes in LV loading on LA physiology and atrial arrhythmogenicity in nineteen pigs following subacute MI. Fourteen pigs were undergone LV unloading with an Impella LVAD at one to two weeks after the MI and five pigs undergone LV overloading-induced aortic regurgitation. Researchers showed that LV unloading with the Impella reduced LA pressures and volumes compared to the measurements obtained in pigs with LV overloading. LVAD placement was shown to decrease nicotinamide adenine dinucleotide phosphate oxidase 2 (NOX2) levels that was elevated after MI in LA tissue. The study concluded that NOX2 and downstream ryanodine receptor modulation may be related to reduced atrial arrhythmias during LV unloading [39].

Hospital mortality rate of CS associated with acute MI (AMI-CS) is nearly 50% due to a decrease in cardiac output, organ hypoperfusion, tissue hypoxia, and cellular damage [41]. Therefore, therapeutic approaches of patients with AMI-CS include ECMO and catheter-based pMCS [42]. In two separate studies, Riehle *et al*. [43, 44] aimed to identify biomarkers and prognostic markers under AMI-CS conditions following LV unloading. Microsphere particles were injected into the left main coronary artery to induce acute MI in pigs and the Impella support was initiated after the onset of AMI-CS. In the initial study, miRNA-200b, which is a hypoxia-induced miRNA, was suppressed during CS and was restored following ventricular unloading [44]. In their second study, biomarker scores of four CS proteins (beta-2-microglobulin, aldolase B enzyme (ALDOB), liver-type fatty acid-binding protein (L-FABP), SerpinG1) and CLIP (Cystatin C, Lactate, IL-6, NT-proBNP) scores were developed to capture organ dysfunction and changes in metabolism to identify high-risk patients for acute CS; however, the scores did not change during the acute phase of CS and LV unloading [43]. These studies suggested that transcriptional changes of miR-200b may occur before noticeable changes in biomarker and CLIP scores [43, 44].

The benefits of veno-arterial ECMO (VA-ECMO) in CS may be limited due to increased afterload to the LV, which can lead to LV dilation, increased LV and LA end-diastolic pressures, thrombosis, and pulmonary edema, compromising myocardial recovery and prolonging lung injury [42]. To mitigate these effects, Mlcek *et al*. [45] used LV unloading with percutaneous balloon atrial septostomy (BAS) in a porcine model of AMI-CS treated with VA-ECMO. In this study, VA-ECMO stepwise protocol (40–80 mL/kg/min) was introduced in female pigs with coronary artery balloon occlusion before and after the BAS was performed. Results showed significantly decreased LV end-diastolic pressure and SV immediately after BAS while the pigs were on VA-ECMO support, indicating that BAS can be used for mechanical unloading [45]. Another study investigated the differences in LV unloading using the Impella and VA-ECMO in female swine with severe CS induced by injecting microspheres in the left main coronary artery [46]. Both devices were successful in improving end-organ blood supply, although VA-ECMO was associated with higher local venous oxygen levels and higher mean arterial pressures (MAPs) and the Impella was linked with decreasing requirements for myocardial oxygen demand. The study concluded that both, the Impella and VA-ECMO, have various benefits and their clinical use should be determined on a patient-to-patient basis [46, 47]. Weil *et al*. [48] induced acute reperfused MI (ARMI) in seven pigs and compared hemodynamic effects and aortic blood pressure of the Impella CP and TandemHeart pLVADs. The Impella CP was placed in the left femoral artery in a swine model of ARMI, while the TandemHeart return line was placed in the right femoral artery with the transeptal cannula inserted *via* the right femoral vein. The Impella CP followed by TandemHeart was placed in four animals, while three animals received the TandemHeart followed by the Impella CP. While both devices had comparable device flow rates and maintained aortic pressure, results showed that the TandemHeart significantly decreased LV SV and LV preload *versus* the Impella CP [48]. A study by Udesen *et al*. [49] aimed to compare vasoactive drugs such as epinephrine, dopamine, norepinephrine, and phenylephrine in a porcine model of AMI-CS supported by the Impella CP device. Study results showed that catecholamines increased LV stroke work and oxygen delivery to the detriment of increasing cardiac work, whereas phenylephrine caused an increase in cardiac work but did not increase oxygen delivery, which warns against using phenylephrine in the treatment of AMI-CS [49, 50, 51].

Briceno *et al*. [52] hypothesized that LV unloading before reperfusion increases collateral blood flow to ischemic myocardium, therefore reducing MI size. To test, adult Yorkshire swine underwent occlusion of LAD artery followed by reperfusion were randomly assigned to have continued occlusion with activation of either Impella CP or VA-ECMO. Mechanical unloading with the Impella CP reduced the infarct size and increased collateral flow index (CFI) compared to unloading with VA-ECMO, which insignificantly impacted these parameters, suggesting clinical importance for the selection of mechanical devices during AMI-CS [52]. Another study using the A-Med LVAD before reperfusion in porcine models discovered reduced infarct size with reduced ET-1 and calcium levels with AMI-CS, suggesting that LV unloading may prevent IRI [53]. Lastly, Saeed *et al*. [54] studied the ability of a soft robotic ventricular assist device (SRVAD) with septal bracing for the management of LV systolic dysfunction. Systolic dysfunction of the LV can lead to mitral valve regurgitation due to increased LV pressures and chamber dilatation. The SRVAD was implanted *via* thoracotomy in 6 adult Yorkshire pigs with induced acute LV systolic dysfunction. In this experiment, SRVAD also successfully eliminated mitral valve prolapse and improved LV wall mobility and function [54], demonstrating the potential of incorporating soft robotics in addressing myocardial damage and cardiac dysfunction [54].

### 2.4 Canine (Dog) Studies

Historically, canines have been used extensively in cardiovascular studies and a substantial reference literature available on their physiology and pathophysiology [84]. Using a canine MI model, Achour *et al*. [55] studied a transvalvular LVAD that was implanted just before reperfusion and after reperfusion and determined that LV unloading before reperfusion reduced infarct size. In another canine model of acute MI induced by ligating the LAD coronary artery, researchers demonstrated that both partial and total support before reperfusion with the Impella CP reduced LV end-diastolic pressure and maintained arterial pressure, which helped avoid hemodynamic instability post-MI [56]. Total LVAD support was associated with improved LV ejection fraction, increased LV end-systolic elastance, and decreased NT-proBNP and prevented subsequent heart failure four weeks post-MI compared with partial Impella support [56].

Tedoriya *et al*. [95] investigated the hemodynamic effects of different coronary artery bypass grafts using IABP or LVAD. Three types of grafts were performed in the canine models: an ascending aorta-coronary bypass graft (ACB), an internal thoracic artery graft (ITA), and a descending aorta-coronary bypass graft (DCB). Blood flow in the LAD coronary artery through these grafts was studied during or in the absence of ventricular assistance. Results demonstrated that the specific combination of the graft type and mechanical device used influenced diastolic flow. For example, the use of LVAD increased diastolic flow in the ACB, ITA, and DAC grafts compared to IABP and the control, underscoring the crucial role of considering hemodynamic features for selecting surgical management [95].

Furthermore, several canine studies have been conducted comparing the hemodynamic benefits of CFVAD *versus* PFVAD pumps [23, 96, 97, 98]. Lim *et al*. [99] developed a three-dimensional (3-D) electromechanical canine model of failing ventricles combined with CFVADs and counter-pulsating VADs to measure the contractile energy consumption of the myocardium and elucidate the effect of these LVADs on myocardial recovery. Although the LV peak pressure decreased to 10% with the PFVAD and 46% with the CFVAD, ATP consumption decreased to only 50% with the PFVAD and 60% with the CFVAD, which was in accordance with similar myocardial recovery observed in clinical trials [99].

## 3. Findings From Small Animal Studies 

Heart failure is associated with unfavorable cardiac remodeling such as cardiac fibrosis, cardiomyocyte hypertrophy or atrophy, apoptosis, autophagy, and inflammation [7]. Ventricular unloading with LVAD support is associated with cardiac ‘reverse remodeling’, a process that may result in partial recovery of cardiac pathology and ventricular dysfunction as summarized in Table 2 (Ref. [24, 29, 35, 36, 37, 38, 39, 45, 46, 47, 48, 52, 53, 54, 57, 58, 59, 60, 61, 63, 65, 66, 67, 71, 73, 80, 85, 87, 89, 90, 92, 93, 94, 95, 99, 100, 101, 102, 103, 104, 105, 106, 107, 108, 109, 110, 111]). The process of ‘reverse remodeling’ involves modifications at the molecular, cellular, tissue, and organ levels in the LVAD supported heart as well as in other organ due to improved cardiac function and increased SV [7, 112, 113]. Mechanical circulatory support is associated with reversion of hypertrophic remodeling of cardiomyocytes [100], improved calcium transients and microvascular density [101], mitochondrial [102], cytoskeletal dystrophin organization [103] and cardiomyocyte regeneration [104], while no change was observed in cardiomyocyte apoptosis [57], no change or increase in myocardial fibrosis and macrophage phenotype switch [58], activation of endothelial cells [59], and increased fibrosis of the coronary adventitia [60]. Numerous small animal (rat or mouse) models have effectively been utilized in these studies aimed to understand the effects of mechanical and volume unloading on the circulatory system, on the failing heart as an organ, as well as on tissue, cellular, and molecular levels.

**Table 2.** **Reverse cardiac remodeling in response to mechanical unloading**.

			Pre LAD	Post VAD	Reference
1. Heart					
	a. Organ level				
		Cardiac function	down	up	[45, 48, 54, 85, 92, 93, 99, 106]
		LV diastolic wall stress	up	down	[38, 45, 89, 95]
		RV pressure overload	up	down	[71, 94]
		Atrial pressure and volume	up	down	[39, 71]
	b. Tissue level				
		Fibrosis	up	down	[36, 37, 58, 63, 66]
		Infarct size	up	down	[24, 36, 37, 52, 53, 90]
		Oxygen consumption/demand	up	down	[46, 47, 90, 99]
		Coronary and microvasculature	down	up	[38, 52, 59, 60, 95]
	c. Cell level				
		Hypertrophy	up	down	[100, 105]
		Atrophy	up	down	[65, 73]
		Calcium transients	abnormal	normalized	[53, 92, 101]
		Apoptosis, necrosis	up	down	[29, 36, 57, 65, 66]
		Inflammation, macrophages	up	down	[29, 58]
		Regeneration	down	up	[24, 87, 104]
	d. Subcellular level				
		Mitochondria			[61, 102]
		Cytoskeleton	disruption	normalized	[63, 67, 103, 106]
2. Other organs					
		End-organ blood flow	down	up	[35, 46, 47, 87, 110]
		Pulmonary function	up	down	[35, 109]
		Clotting	abnormal	improvement	[80, 107, 108, 109, 111]

LAD, left anterior descending; VAD, ventricular assist device; LV, left ventricular; RV, right ventricular.

### 3.1 Rat Models 

To investigate the pathophysiology of mechanical unloading in rats, researchers first conduct a transverse aortic constriction (TAC) procedure to induce cardiac hypertrophy and heart failure. Subsequently, LV unloading is simulated by a heterotrophic heart transplant (hHT_x_), in which a donor heart is connected in parallel with a recipient heart [7, 8]. Schaefer *et al*. [114] introduced a novel rat model, in which hHT_x_ is performed 3–6 weeks after a TAC procedure. Based on histology and echocardiographic measurements, the authors suggested that a TAC procedure in rats mimics the human physiology of moderate to severe heart failure with pressure overload and dilated LV [114]. To compare a rat model with humans, gene expression analysis of rat atrophied hearts following hHT_x_ was performed and the results were compared with failing and unloading human hearts [61]. The study demonstrated that rat hearts following unloading *via* hHT_x_ have reduced expression of NADH-DH, cytochrome c oxidase (COX or mitochondrial complex IV), acetyl-CoA synthetase, and myoglobin, but increased expression of major histocompatibility complex 1 (MHC1), MHC2, and heat shock protein 70 (HSP70). All there changes in rat simulated changes observed in human failing heart after LV unloading [61]. These changes in genetic markers following mechanical unloading indicate that hHT_x_ in rats is a useful model to study the molecular mechanisms of LV unloading [61].

The usefulness of rat animal models in MCS-associated research extends to engineered heart tissue (EHT). Researchers reported that pouch-like EHTs from neonatal rat hearts injected into adult rats can simulate biological VADs *in vivo* [62]. It has been demonstrated that over time, the EHT grafts bear structural and functional semblance to native cardiomyocytes. Furthermore, Shen *et al*. [63] created a novel *in vitro* EHT model as a representation of myocardial reverse remodeling. The platform was created by culturing decellularized porcine myocardial sections with primary cardiomyocytes and fibroblasts isolated from neonatal rat ventricular myocardium or with cardiomyocytes derived from human-induced pluripotent stem cells (hiPSC) [63]. Characterization of EHTs demonstrated gradual normalization of stress-free tissue length after mechanical unloading and suggested crucial roles of actomyosin contraction in cardiomyocytes and activity of fibroblasts in reverse remodeling after mechanical unloading [63]. Reverse remodeling in the human failing heart involves numerous kinases such as extracellular signal-regulated kinases (ERKs), mitogen-activated ERKs (MEKs), protein kinase B (known as AKT), c-Jun N-terminal kinases (JNKs), and p38-mediated signal transduction pathways with significant decreases in ERK and AKT kinase activity and increases in glycogen synthase kinase-3 beta (GSK-3β) activity after LVAD implantation [105]. A study by Baba *et al*. [105] found similarities in signal transduction pathways between rat and human hearts and validated ERKs, AKT, and GSK-3β modifications using cyclic strain in neonatal rat cardiomyocytes *in vitro* and tensile stretch with LV balloon in a mouse heart perfused with Langendorf system. Based on the results, MEK/ERK, AKT, and GSK-3β signaling pathways are highly sensitive to pressure changes from LVAD support and serve as potential therapeutic targets [105].

While mechanical unloading leads to favorable cardiac reverse remodeling, VAD support often causes cardiac atrophy, one of the most common adverse effects of MCS devices [100]. Cardiac unloading is associated with decreased expression of α*MHC* and *SERCA*, while levels of β*MHC* and *ANP *are markedly increased, suggesting activation of a cardiac ‘fetal gene program’ linked to deteriorating cardiac function [64]. In rat hHT_x_ models, investigators explored whether reduced ventricular mass following prolonged LV unloading in hHT_x_ rats was attributable to cardiac atrophy (reduce in) or apoptosis based on cytoplasmic indices, TUNEL, caspase-3 assay, and electron microscopy results [65]. They ultimately reported that the main cause of ventricular mass reduction is cardiac atrophy as opposed to apoptosis and suggested that myocardial mass reduction in LVAD-unloaded hearts may be irreversible. Other rodent studies have investigated the use of medications to reduce cardiac apoptosis in unloaded rat hearts and found that neither carvedilol nor metoprolol offered any significant benefit to reduce apoptosis when administered during LV unloading, metoprolol treatment resulted in reducing cardiac fibrosis [66].

### 3.2 Mouse Models 

In mice, a TAC procedure has been conducted to induce a pressure-overload state, and the subsequent removal of aortic constriction is used to simulate LV unloading [67]. Results demonstrated that TAC-induced pressure overload causes myocardial fibrosis, collagen fiber disarray, and cardiomyocyte disorganization and hypertrophy. Upon LV unloading, electron microscopy revealed improved cellular maladaptation in myofibroblasts and cardiomyocytes, while the disarray of collagen fibers has remained [67]. Another group used a transgenic mouse model to generate a dilated heart failure phenotype that expresses inflammatory gene TNF receptor-associated factor 2 (TRAF2) [106]. To induce reverse cardiac remodeling, these heart failure mice were treated with doxycycline for 4 weeks. At the end of treatment, only 58% of heart genes were normalized, despite these mice had restoration of contractility, heart structure and LV size. Moreover, these mice had increased susceptibility to subsequent hemodynamic injury, illuminating a discrepancy between genetic and functional recovery during reverse remodeling of the heart. The study also demonstrated that the mice treated with doxycycline for 8 weeks achieved a greater percentage of heart failure gene normalization and lesser susceptibility to hemodynamic injury [106], suggesting associations between genomic recovery and myocardial vulnerability.

## 4. Animal Models Used for Devise Improvement and Surgical Training 

As VAD implantation becomes a mainstay treatment for patients with heart failure, animal models are beneficial for surgical training to minimize human error during surgery [84, 115]. Anatomical differences between animal models and their applicability to humans provides a key information for selecting a surgical approach in patients. For example, the large vasculature in cows allows for easier device cannulation when compared to other animal models, and the similarities between the human and porcine aortic valves allows for important translational research [84].

HeartWare, Inc. developed a miniature LVAD with wide-blade rotors, and the device was inserted into a jersey calf *via* left thoracotomy and assessed the performance over 30 days [116]. Researchers observed no adverse effects over the course of the study and deemed the device successful, supporting the feasibility for use in animal models and potentially in human patients [116]. Another study further assessed a small left thoracotomy for the HeartWare LVAD implantation in 10 cows [117]. The surgeries and subsequent LV unloading were successful in all animals, thereby providing further support for the use of minimal invasive surgeries for device insertion that do not require sternotomy. Two separate groups have developed animal models for practicing implantation of CFVADs. Zhang *et al*. [118] described three surgical practice sessions using a CFVAD pump in five pigs, which improved the technical performances of surgeons based on self-evaluation and evaluation from an experienced surgeon. The team noted improved surgical performance in the third session in comparison to the first based on improved operative times and success of implantation. Another porcine model was described by Robinson *et al*. [68] in which an *ex vivo* porcine heart along with the great vessels was held in a heart cradle of a simulator during training of minimally invasive LVAD implantation.

## 5. Animal Models Used for Studying VAD Complications

LV devices have historically been suitable only for patients with larger body sizes and have required more invasive surgical insertion [116]. Compared to the first LVADs implanted in the 1960s, current LVADs have miniaturized, thus surgical approaches have progressed from sternotomy to minimally invasive techniques [119]. With improvements in LVADs and the limited availability of donor hearts for transplantation, LVAD therapy has become more prevalent within the last decades, surpassing the number of heart transplantations in patients with end-stage heart failure [69]. Achieving long term survival in end-stage heart failure recipients is remarkable with advanced MCS devices; however, success is constrained by high-risk complications such as bleeding, thromboembolic and acquired coagulopathy, events necessitating anticoagulant and antiplatelet therapies [120, 121]. Other complications impeding longer term survival of LVAD-supported patients include stroke, infections, and end-organ dysfunction including RV, kidney, and hepatic failure [70]. Some of adverse events are related to MSC device malfunctions, while other complications are related to surgical techniques or perioperative management [122]. Animal studies have also been widely used to investigate LVAD-related complications and improve surgical approaches.

### 5.1 Studies on Clotting Differences Using Multi-Species Modeling

Clotting and inflammatory parameters vary between species, and animal models that adequately mimic parameters of human blood clotting are important for preclinical studies. Hence, results of a study assessing tissue factor and partial thromboplastin phospholipid clotting parameters in whole blood of different species (humans, calves, goats, and pigs) using thromboelastography demonstrated significant differences in extrinsic and intrinsic clotting parameters among the four species studied [107]. Specifically, the maximum clot firmness was significantly higher in calves and goats compared to that of humans. These differences are important to consider when studying mechanical devices in animal models since the devices interact with blood and can potentially lead to complications such as coagulation, bleeding, and thromboembolism.

### 5.2 Modeling of Post-LVAD Right Heart Failure and Cardiac Atrophy

A post-LVAD implantation RV failure is one of major complications that is associated with high morbidity and mortality [123]. Management of RV failure was investigated by Saito *et al*. [71] in a goat model. In this study, seven goats were implanted with an ECMO in combination with a centrifugal pump LVAD and then an RV failure was induced by PA banding, followed by a balloon atrial septostomy. Balloon atrial septostomy is a procedure, usually used for treating primary pulmonary hypertension, that expands a foramen ovale or atrial septum defect to unload the RV. The balloon atrial septostomy demonstrated promise as a solution for RV failure following LVAD implantation since the interatrial shunt yielded increased LVAD flow, increased mean arterial pressure, and decreased right atrial pressure [71].

Prolonged LVAD support has been shown to induce cardiac atrophy, another important MCS complication and the lack of significant improvement in myocardial function in LVAD patients has been linked to cardiac atrophy [72, 124]. Pokorný *et al*. [73] demonstrated the mitigating effects of a spring expander on cardiac atrophy in failing rat hearts [73]. In this study, rats with failing hearts were introduced with ventricular unloading through hHT_x_ and then induced with heart failure *via* an aorto-caval fistula creation. Cardiac atrophy was measured by the ratio of the transplanted heart weight to the control heart weight. The spring expander, which enhanced isovolumic loading without obstructing LV function, was shown to reduce cardiac atrophy [73].

## 6. Preclinical Animal Models of LVADs Intended for Pediatric Patients

The development of MCS devices for long-term use in pediatric patients remains challenging, especially in children with a body surface area <0.7 m^2^ [74]. When selecting MCS for young children requiring cardiac intervention, physicians should consider specific anatomical and physiological features relevant to cannulation, circuit configuration, anticoagulant management, timing of intervention, and selection of optimal surgical candidates [75]. Despite advances in LVAD models assessing these features and risks relevant to young patients, researchers recommend further exploration in animal models.

The first VAD device approved by the Food and Drug Administration (FDA) for long-term univentricular and biventricular support in pediatric patients is the Berlin Heart EXCOR (Berlin Heart GmbH, Berlin, Germany) [76]. Several studies involving animal modeling have been conducted to evaluate the increased risk of hemolysis and thrombosis associated with the EXCOR device alone and the feasibility of EXCOR use with additional assist devices [77]. For instance, Wermelt *et al*. [78] studied the practicality of combining EXCOR and Novalung, an interventional lung assist device (iLA, GmbH, Hechingen, Germany) in 10-kg pigs with a 10 mL blood pump and 30-kg pigs with a 30 mL pump. Researchers ultimately demonstrated that a PFVAD combined with iLA use is feasible, especially in pediatric patients with hemodynamic and respiratory failure [78].

The National Heart, Lung, and Blood Institute (NHLBI) launched the Pediatric Circulatory Support program for developing and preclinical testing of novel MCS devices designed specifically for infants and patients up to 2 years of age [79]. The Pediatric Circulatory Support program granted funding to several institutions to encourage the development of pediatric VADs. Penn State was one such institution which developed the Penn State Infant VAD with a 12–14 mL SV and pneumatically actuated pump and tested in juvenile lambs with a mean body weight of 23.5 ± 4.1 kg for long-term support (5 to 41 days; mean 26.1 days). The study results were consistent with the results of adult VAD testing in animals, demonstrating minimal device thrombi which were further reduced by introduction of the custom valve [80]. In a study by Lukic *et al*. [81], the Penn State Infant VAD was implanted in 12 lambs of 18–29 kg *via* left thoracotomy with cannulation of the LV and descending aorta without cardiopulmonary bypass and the pump was implanted subcutaneously in the left flank. Results of this study demonstrated that the Penn State Infant VAD has minimal thromboembolic risks, thereby allowing for lower levels of anticoagulation during MCS. The Pediatric Circulatory Support Program also led to the development of the PediPump (Cleveland Clinic) and PediaFlow (University of Pittsburgh-lead consortium). An ovine model was utilized in the development of the PediPump VAD, and researchers also noted trivial VAD-associated hemolysis [82]. An ovine model was also used to study the second-generation PediaFlow device (PF2), which differed from the first generation by increased flow rates and decreased device volume [108]. Given the increased risks of thromboembolism in individuals with LVADs, researchers aimed to investigate platelet activation in three sheep implanted with the PF2 device and monitored them for 16, 30, and 70 days. In this study, increased platelet activation after surgery was seen in each sheep, eventually returning to pre-VAD levels. For example, one sheep experienced a significant platelet activation on postoperative day 16 and this event was explained by erroneous VAD pump stoppages and associated regurgitant flow. Another sheep had elevations of platelet activation on postoperative days 13 and 20, which may have been related to impeller scratches identified at necropsy. Five additional sheep with Sham surgery also demonstrated postoperative platelet activation that returned to baseline levels in about 2.5 weeks. Overall, these studies demonstrated the biocompatibility of the PF2 device in ovine models [108].

A juvenile lamb model was used in the study of the PediVAS device intended for kids between 3 and 20 kg to assess the device biocompatibility and risk factors associated with surgical implantation. Researchers conducted three studies evaluating a device functionality over 30 days and three subsequent studies evaluating device functionality over 8 hours [125]. BiVAD and LVAD devices were successfully implanted *via* thoracotomy, and all six juvenile animals survived study periods without major complications. Although researchers noted ring thrombus and small deposits on the devices at necropsy, these finding have been noted as irrelevant to device safety and functionality. A juvenile sheep model was also used in another study comparing the Jarvik 2000 and CircuLite pediatric VADs for 26.7 ± 19.6 days [126]. The study noted initial difficulties including leg injury, damage to the myocardium and poor positioning of the device during implantation, bleeding, lengthy operative time and maintaining respiratory function in juvenile animals, highlighting the clinical issues faced by MCS implementation in pediatric patients. The subsequent experiments were able to solve some of these issues by changing anticoagulation and using sling support to mitigate leg injury [126]. Another study examined the use of the NIPRO-LVAD, a device intended for use in children or adults with small body size, in four Shiba goats followed for 30 to 90 days. Absence of significant thrombosis on the devices at necropsy of the goat model suggested biocompatibility of the device and promising potentials for use in pediatric patients [109].

Neonatal animal models have also been used to study pediatric VADs. Researchers induced right heart dysfunction in 3-week-old lambs (8.6 kg) *via* right ventriculotomy and implanted the MEDOS trileaflet-valved pediatric RV assist device (SV = 9 mL) to support lambs for 6 hours [127]. This successful animal modeling has led to the implantation of VADs approved for adults in children and infants with heart failure. For example, a group of researchers altered the flow parameters of the TandemHeart (CardiacAssist, Pittsburgh, PA), a VAD approved for adult use, and the modified VADs were implanted in seven anesthetized piglets (6–14 kg) *via* median sternotomy [128]. In this study, a recirculation shunt was used to reduce flow volumes and the piglets were successfully supported by the devices for 4 hours. Following these preclinical studies, the modified TandemHearts were successfully implanted into a 10-month-old 10-kg infant with restrictive cardiomyopathy for 5 days and a 5-year-old 18.7-kg child with myocarditis for 13 days until heart transplantation occurred [128].

The IABP device is an additional form of mechanical support that has been successfully used in larger children and adolescents but has limited applicability in smaller children [75]. The efficacy of ECMO, IABP, PFVAD, and CFVAD was compared in 47 Yorkshire piglets with coronary artery ligation [110]. The study demonstrated improvements in LV blood supply/demand ratio during PFVAD, CFVAD and ECMO support, an improved global myocardial blood supply/demand ratio during PFVAD and CFVAD support, and diminished pulsatility during ECMO and CFVAD support, suggesting different mechanisms for each type of pediatric VAD. Based on these data, the authors established an important resource profile for selection of pediatric patient for each type of device studied [110].

Questions arise regarding pediatric LVAD explant protocol on how and when is optimal to wean patients off the devices. One group of researchers assessed LVAD weaning parameters using a pneumatically driven pediatric PFVAD using *in vitro* mock circulatory loop (MCL) and compared slow *versus* quick-filling conditions over 4 different SVs [111]. The study results demonstrated that reducing SV from 100% to 60% is superior to beat reduction, while a quick filling is superior to slow filling given that the latter is associated with increased thrombus formation [111]. In summary, the pediatric population presents challenges for MCS devices due to specifics in anatomy and physiological parameters as well as surgical, post-LVAD and anticoagulation management. Currently, ECMO devices are predominantly used in pediatric patients as a bridge to recovery or transplant; however, the devices carry the risk of postoperative complications. Various VADs have been developed and tested in juvenile animal models, including pigs, lambs, and sheep, with promising results in terms of biocompatibility and functionality.

## 7. Conclusions 

Despite differences in anatomical, physiological and clotting parameters, animal models have played a crucial role in development and advancement of MCS devices and improving the management of heart failure in both adult and pediatric patients. As LVADs become more common in heart failure management, animal modeling offer significant usefulness and advancements to address the limitations in MCS devise size, durability, hemo and biocompatibility.
